# Diarrheagenic *Escherichia coli* Associated with Acute Gastroenteritis in Children from Soriano, Uruguay

**DOI:** 10.1155/2018/8387218

**Published:** 2018-10-24

**Authors:** Vivian Peirano, María Noel Bianco, Armando Navarro, Felipe Schelotto, Gustavo Varela

**Affiliations:** ^1^Bacteriology and Virology Department, Hygiene Institute, Medicine Faculty, Universidad de la República, Uruguay; ^2^Mercedes Hospital Laboratory, State Health Services Administration (ASSE), Uruguay; ^3^Public Health Department, Medicine Faculty, UNAM (Universidad Nacional Autónoma de Mexico), Mexico City, Mexico

## Abstract

**Introduction:**

Acute diarrheal disease still deserves worldwide attention due to its high morbidity and mortality, especially in developing countries. While etiologic determination is not mandatory for management of all individual cases, it is needed for generating useful epidemiologic knowledge. Diarrheagenic *Escherichia coli* (DEC) are relevant enteropathogens, and their investigation requires specific procedures to which resources and training should be dedicated in reference laboratories.

**Methodology:**

Following the hypothesis that enteric pathogens affecting children in towns located in the interior of Uruguay may be different from those found in Montevideo, we conducted a diagnostic survey on acute diarrheal disease in 83 children under 5 years of age from populations in the south of the country.

**Results:**

DEC pathotypes were the only bacterial pathogens found in diarrheal feces (20.48%), followed by rotavirus (14.45%) and enteric adenovirus (4.81%). Atypical EPEC (aEPEC) was the most frequent DEC pathotype identified, and unexpectedly, it was associated with bloody diarrheal cases. These patients were of concern and provided with early consultation, as were children who presented with vomiting, which occurred most frequently in rotavirus infections. aEPEC serotypes were diverse and different from those previously reported in Montevideo children within the same age group and different from serotypes identified in regional and international studies. Enteroinvasive (EIEC) O96 : H19, associated with large outbreaks in Europe, was also isolated from two patients. Antibiotic susceptibility of pathogenic bacteria identified in this study was higher than that observed in previous national studies, which had been mainly carried out in children from Montevideo.

**Conclusion:**

The reduced number of detected species, the marked prevalence of aEPEC, the scarce resistance traits, and the diverse range of serotypes in the virulent DEC identified in this study confirm that differences exist between enteropathogens affecting children from interior towns of Uruguay and those circulating among children in Montevideo.

## 1. Introduction

Infectious diarrhea causes almost 500,000 deaths per year, especially among children up to five years of age from Asia, Africa, and Latin America [1, 2]. Incidence varies between countries and regions, due to a number of recognized factors, such as the socioeconomic group, nutritional status, access to safe water sources, wastewater disposal, food safety, electricity supply, refrigeration of food, and close contact with animal reservoirs of potential pathogens. Infectious diarrheal diseases are particularly prevalent in younger children from low income homes [3–5].

Diarrheal disease is very important due to its high morbidity and mortality. Attention must also be given to its links with malnutrition and to the high cost of medical attention that impacts an already burdened health system in many developing countries [6]. Severe cases and related complications often require specialized care, which includes diarrheal diseases characterized by severe dehydration (found in cholera cases), bloody diarrhea caused by *Shigella*, haemolytic-uremic syndrome (HUS) associated with infection by Shiga toxin-producing *E. coli* (STEC), Guillain–Barré Syndrome (GBS) linked to *Campylobacter,* and invasive illness by *Salmonella* or acute abdominal pain due to mesenteric adenitis and *Yersinia enterocolitica* [7, 8].

Laboratory investigation of all potential diarrheal agents presently involves complicated and expensive procedures, and it is not usually required or performed to manage individual cases [9, 10]. However, control measures to combat acute diarrheal disease of children in primary care settings cannot be adequately oriented if predisposing conditions, etiologic agents, and their epidemiologic-spread profile are not fully known and available to health care decision-makers [2, 5].

Diarrheagenic *E. coli* (DEC) is a group of strains that do not form part of the human intestinal microbiota but can be transmitted from food or infected humans to susceptible children and adults resulting in a range of disease that can be very serious and frequent. Several overlapping virulent types that are capable of gene transfer include enterotoxigenic *E. coli* (ETEC), enteropathogenic *E. coli* (EPEC), enteroaggregative *E. coli* (EAEC), Shiga-toxin-producing *E. coli* (STEC), and enteroinvasive *E. coli* (EIEC). Diffuse-adherent *E. coli* DAEC or adherent-invasive *E. coli* AIEC is also a potential member of DEC that requires further study [11, 12].

For many years, our workgroup has participated in the surveillance of acute diarrheal disease in Uruguayan children [13–16], and we confirmed that EPEC is the most prevalent DEC pathotype locally. Shiga toxin-producing *Escherichia coli* (STEC), mainly non-O157, EAEC, and ETEC were also identified as being present locally, and EIEC was confirmed but less frequently [13, 14].

Most Uruguayan DEC studies (and more general studies concerning diarrheal disease and pertinent agents) have been conducted in urban Montevideo [13–16] and have provided useful information. However, these studies need to be complemented with studies from smaller towns and rural areas where the epidemiology, spread, distribution, and characteristics of enteropathogenic microorganisms can vary [17].

This study deals with the etiology of acute diarrhea in children and intends to overcome the mentioned weaknesses of existing knowledge, by focusing on DEC detection in children from small towns in the interior regions of Uruguay and on characterization of isolates.

## 2. Materials and Methods

The study period ran from October 2012 to March 2015.

### 2.1. Approvals and Consent

The study was approved by the Ethics Bureau of Medicine Faculty, UdelaR, and by the Mercedes Hospital Committee. An informed consent was obtained from each child´s parent, following the explanation of the study and procedures.

### 2.2. Sampling and Data Recording

We examined stool samples (*n*=83) from children up to 5 years of age who suffered acute community diarrhea, defined as three or more discharges within 12 hours, or just one liquid or semiliquid stool including mucus, pus, or blood. The children were brought to the attention of health services of small- or medium-sized towns; most of them in Mercedes, Soriano. Children with persistent diarrhea, patients receiving antibiotics, or those who had been hospitalized within 30 days prior to the onset of diarrhea were excluded.

A single stool sample was obtained from each child through spontaneous defecation and was collected in a sterile, wide-mouth plastic container. Part of the sample was transferred with a sterile swab to a tube of semisolid Cary–Blair transport medium (C-B) (HIMEDIA®Laboratories).

Data regarding the symptoms of the disease, macroscopic stool aspect, nutritional and hydration status, therapy administered, and potential infected contacts, were collected for each patient as carefully as possible.

### 2.3. Microbiological Analysis of Stools

The detection of rotavirus and adenovirus antigens was performed in the Mercedes Hospital Laboratory by the immunochromatographic technique, according to the manufacturers´ instructions (RIDA Quick Norovirus and RIDA Quick Rotavirus/Adenovirus Combi-Biopharm AG, Darmstadt, Germany). Both parts of the sample (with and without C-B transport medium) were immediately sent to the Bacteriology and Virology Department, at the Institute of Hygiene. Identification of enteric pathogens was conducted there as previously described [13, 14].

Following macroscopic observation to identify abnormal components (blood, pus, or mucus), two slide smears were prepared from feces without the transport medium: one stained with methylene blue for detection and gross quantification of fecal leucocytes, and the other one stained with the modified Gram technique (Ziehl's fuchsin diluted 1/10 instead of Safranin as counterstain) to discover spiral forms, suggestive of *Campylobacter*.

Enrichment broths for STEC, *Salmonella, Yersinia*, and selective-differential plate media for isolation of *Salmonella, Shigella*, *Yersinia enterocolitica, Campylobacter,* and *E. coli* pathotypes were inoculated from both parts of the stool sample (with and without C-B transport medium) to optimize pathogen recovery. Dense feces were diluted in saline solution.

The enrichment broths used were Tetrathionate broth (TT) for *Salmonella,* cefixime-tellurite trypticase Soy Broth (CT-TSB) for STEC, and peptone sorbitol bile broth (PSB) for *Yersinia*. Plating media were MacConkey Lactose and Sorbitol MacConkey (SMAC), mainly employed for the isolation of DEC, *Salmonella*-*Shigella* agar (SS), and Skirrow selective medium for *Campylobacter*. *Yersinia enterocolitica* was selected on MacConkey agar or cefsulodin-irgasan-novobiocin (CIN) agar. The commercial sources for most of the culture media were Difco®, Becton-Dickinson, and Oxoid® Ltd., Basingstoke, Hampshire, UK, while Sigma-Aldrich® and bioMérieux®, Marcy l'Etoile, France, provided added chemical or antimicrobial mixes.

One gram or 1 ml stools were inoculated in 10 ml liquid enrichment broths. Subculture from CT-TSB was performed on SMAC before 18 hours incubation, after 24 hours from TT to SS for *Salmonella* and after 21 days incubation at 4°C–8°C on MacConkey or CIN media from PSB. Incubation was kept at 35°C–37°C for most media, at 28°C for *Yersinia*, at 43°C for TT broth, and for Skirrow plates included in microaerophilic environment.

Classical phenotypic tests were employed to identify *Salmonella, Shigella, Yersinia*, and *Campylobacter*. Occasionally, it was necessary to use the API 20E system (bioMérieux®, Marcy l'Etoile, France) or Vitek 2 and MicroScan/AutoScan® equipment for completing the identification of isolates.

### 2.4. Investigation of DEC Pathotypes

Suspected *E. coli* colonies on MacConkey or SMAC plates were studied by PCR screening [14, 16, 18] following a two-step process:

- Firstly, gene-specific PCR assays were performed to detect DEC pathotypes (Table 1) in DNA extracted from the confluent growth zone of spread plates and from several 10-colony pools taken from primary or subculture plates. The pools included sorbitol negative, sorbitol positive, and lactose-positive bacteria. Individual colonies were kept at 4°C for further studies.
- A confirmation step followed to amplify sequences of DNA extracted from slant cultures obtained from individual colonies of positive pools. This was not always possible, due to loss of viability of some saved colonies.

For DNA extraction, bacterial cultures suspended in Milli-Q® water and heated in boiling water for 5 minutes. After 10 min at 4°C, they were centrifuged at 13,000 rpm for 10 min, and the supernatant containing released DNA was kept at −20°C until use.

Amplifications were performed in reaction volumes of 25 *µ*L containing 0.2 mM dNTPs, 0.2 *µ*M primers (SBS Genetech Co, Ltd), 10 mM Tris-HCl, 2 mM MgCl_2_, 1.5 U Taq polymerase (HybriPol Bioline, UK), and 2.5 *µ*L crude template DNA. The thermocycler used was a GeneAmp 2700 (Applied Biosystems®, California, US).

Conditions were similar for all reactions, consisting of 94°C initial denaturation for 5 minutes, followed by 30 cycles of 1 min at 94°C followed by different annealing temperatures for 1 min and a further 1 min at 72°C. The final extension period was set at 72°C for 10 min. PCR products were visualized with ethidium bromide staining after electrophoresis in 2% agarose gels in 0.5X TBE buffer.

The first PCR screenings were performed with *stx*1/*stx*2 and *eae* primers focusing on the selection of STEC or EPEC DEC. DNA yielding positive *eae* and negative *stx*1/*stx*2 PCR results was then examined with *bfp* primers to differentiate tEPEC from aEPEC. Negative *eae* and *stx*1/*stx*2 extracts were examined with pCVD432 primers for plasmidic EAEC sequences, *ipa*H primers for detecting genes coding the invasion plasmid antigen of EIEC (and *Shigella)*, and with PCR tests for *eltA* and *estA* genes of ETEC labile and stable enterotoxins.

The primer sequences, annealing temperatures, expected sizes of PCR products, and information sources can be seen in Table 1 [16–22].

Isolates selected as presumptive DEC were biochemically tested to confirm that they belonged to the *E*. *coli* species. Serotyping and antimicrobial susceptibility assays were performed. Pathotypes were confirmed, and data were added to strains identification.

Serotypes were determined at the Universidad Nacional Autónoma de Mexico, using Ørskov and Ørskov′s agglutination assay, 96-well microtiter plates, and rabbit serum (SERUNAM) obtained against 187 somatic antigens and 53 flagellar antigens of *E*. *coli*.

The disc diffusion method was employed as recommended by Clinical Laboratory Standards Institute (CLSI standards) for determining antimicrobial susceptibility of all confirmed DEC isolates [23]. Employed discs (Oxoid® Ltd., Basingstoke, Hampshire, UK) contained ampicillin, cefradine, cefoxitin, cefuroxime, ceftriaxone, ceftazidime, sulbactam-ampicillin, imipenem, meropenem, ciprofloxacin, trimethoprim-sulfamethoxazole, nalidixic acid, gentamicin, and amikacin. Vitek® or MicroScan® systems were used for confirmation when required.

### 2.5. Data Analysis

Statistical analysis was performed by Epi-Info 2000 software developed by PAHO (Pan American Health Organization). When comparing relative frequencies, the chi-square test was used for establishing or discarding a link between qualitative variables. Fisher's exact test was used if sample sizes were small. A *p* value <0.05 was regarded as statistically significant.

## 3. Results

Forty female and 43 male infants (*n*=83) were studied, aged from 20 days to 5 years; the average age was 10 months.

All children showed an adequate nutritional status and hydration level upon onset of acute diarrhea. Other basic clinical data of the children with diarrhea caused by a single enteropathogen are shown in Table 2. Ongoing diarrhea was watery in 24 children (28.91%), semiliquid in 28 (33.75 %), mucoid in 26 (31.32 %), and blood-stained in five (6.02 %).

Cases occurred throughout the year, with higher frequency in late spring and summer.

### 3.1. Number and Types of Detected Enteropathogens

One or more potentially pathogenic enteric agents were identified in 30 of the 83 children (36.14%).

There were 33 enteropathogens identified: DEC, 17 (20.48 %); rotavirus, 12 (14.45%); and adenovirus, 4 (4.81%). DEC distribution was as follows: aEPEC (*eae+, bfp-*, *and stx-*) in 13 children, EIEC (*ipa*H+) in 3, and STEC (*eae+, stx*2+) in one child. Neither ETEC nor EAEC sequences were detected. Three children showed coinfections: aEPEC and rotavirus in two cases and aEPEC and adenovirus in one. Viruses were found as single diarrhea-associated pathogens in 13 children and DEC in 14 cases.

Individual colonies were available for further studies in 13 of the 17 samples in which PCR yielded positive results for DEC. This could not be done with the 4 other DEC suspected plates.  Table 3 shows the pathotypes and serotypes of recovered DEC isolates.

Recovered EIEC isolates (*n*=2) were lactose and lysine-decarboxylase positive, motile, and indol negative. API 20E identification code was the same for both (5104572).

No *Salmonella, Yersinia enterocolitica, Shigella,* or *Campylobacter* isolates were recovered. Significant presence of fecal leucocytes (++ or +++) was only observed in smears from 3 children: 2 with presumptive EIEC and one with confirmed aEPEC. Microscopic examination did not show any spiral bacteria suggestive of *Campylobacter*.

Clinical presentation of cases as related to etiology is shown in Table 2. Diarrhea was more frequently liquid in children from which a pathogen could be identified (16/27 = 59.3% vs 7/53 = 13.2%). Bloody diarrhea was significantly associated with aEPEC etiology: 3 out of 5 children with bloody feces (4, 16, and 35 months old) were aEPEC positive, as compared with 10 of 78 with nonbloody diarrhea (*p* < 0.05**)**. In those 3 cases, there was no virus coinfection.

Rotavirus-infected children presented with vomiting more frequently (46.2%) than aEPEC-positive patients (10%), as shown in Table 2. This difference was not significant (*p* > 0.05). Regarding rotavirus vaccine status, none of the children had been vaccinated at the time of entering the study.

### 3.2. Antimicrobial Susceptibility

Most aEPEC studied strains showed some antibiotic resistance, with ampicillin, cefradine, sulbactam-ampicillin, and trimethoprim-sulfamethoxazole resistance being detected, as shown in Table 3. Two strains were resistant to three mentioned compounds, and 1 to two of them. Both EIEC strains and the single STEC isolate were susceptible to all assayed antimicrobials.

## 4. Discussion

The main observation in this study was that DEC, and especially aEPEC, were the most frequent pathogens found in this group of children, who lived in small towns of southern Uruguay. Rotaviruses were also frequently detected.

All recovered EPEC isolates were classified as atypical, due to the lack of *bfp* plasmidic genes as revealed by negative PCR results [24]. Atypical EPEC had been thought to be less virulent than tEPEC strains; however, it has not been proven that they are less pathogenic. In addition to virulence factors coded in LEE, intimin, Esp (*E. coli* secreted proteins), Tir (translocated intimin receptor), and T3SS (type 3 secretion system), they can express EAST1 (enteroaggregative heat stable toxin 1), E-hly (EHEC-enterohemolysin), Afa (afimbrial adhesin), and many others. Variants of intimin and other components are usually different between tEPEC and aEPEC subtypes, as are O and H antigens defining serotypes. aEPEC is a heterogeneous group of strains with diverse virulence profiles that may have acquired LEE through horizontal transfer or may have come from tEPEC that have lost the EAF plasmid [25–27]. Some strains seem to show more genetic similarity with STEC cell lines than with tEPEC. An aEPEC strain can be a STEC bacterium that has lost phages that code Shiga toxins. STEC and aEPEC have other antigenic and virulence traits in common, for which their relationships deserve attention and analysis in terms of molecular epidemiology. However, clinical isolates of aEPEC from patients in Australia and New Zealand [26] did not seem to derive from STEC or from tEPEC, and their study suggested that type I fimbriae or other adherence structures that are similar in function to bfp may contribute to their virulence.

Fecal leucocytes are seldom found in EPEC infections. However, more sensitive approaches may disclose intestinal inflammatory features or blood contents in diarrheal episodes associated with EPEC [28, 29]. In our study, a significant association was seen between aEPEC infection and bloody diarrhea; aEPEC were present in feces of 3 out of 5 children with bloody diarrhea, a clinical presentation causing concern for parents and health workers. Two of those three strains could be serotyped: O137 : H6, which was reported as an aEPEC isolate from children's feces in Denmark some years ago [30] and O166 : H21 serotype that was previously isolated by other workers as a STEC pathotype strain [31]. Our O166 : H21 isolate was obtained from a child who underwent surgery due to intestinal intussusception, a condition not easily distinguishable from HUS. This is noteworthy because STEC bacteria can lose phages-coding Shiga toxins even during laboratory subcultures and are defined as EHEC-LST [32, 33]. Complete sequencing of these and other aEPEC isolates recovered from children with bloody diarrhea may eventually disclose their genetic relation with STEC strains.

Atypical EPEC have been recovered from children's diarrhea in countries and population groups of middle to high socioeconomic level [34, 35]. Typical EPEC strains are still prevalent in poor regions of sub-Saharan Africa [36], but in other developing areas, aEPEC predominates as seen in developed countries [37]. In America, tEPEC (as defined through classic serogroup determination) was prevalent some decades ago, mainly in developing regions [13, 15, 38]. More recent surveillance work has revealed that aEPEC are more frequent than tEPEC in high-income and also in low income populations and regions [39–45].

In Uruguay, tEPEC and aEPEC still cocirculated 15 years ago among poor children [16], but aEPEC are prevalent in recent years both in children of high and low socioeconomic groups, as shown in this study and in another study performed using identical methods, that included children from high-income households [14].

It is important to highlight the great diversity of serotypes identified in this study that are also different from those found in the aforementioned local studies, and from aEPEC serotypes reported in other countries or regions [42, 46, 47]. However, most of the isolated serotypes and serogroups in this study have been reported as aEPEC or STEC present in animals or food of animal origin that are potential sources of human infection, except those from the O184 serogroup, that may represent a novel finding of diarrhea-associated *E. coli* bacteria that deserves further analysis [30, 31].

Atypical EPEC can have an animal reservoir, are adapted to human and animal hosts, and require particular attention, as well as STEC, when food-borne infection is suspected [24, 30, 48, 49].

Only one O145 STEC strain was identified. STEC isolates are not common in Uruguayan children, even in bloody diarrheal disease [50]. They seem to occur more frequently in children from high or middle-high socioeconomic groups and in small towns outside Montevideo [7, 14, 17, 51, 52]. Non-O157 STEC (O26, O145, and others) are the STEC groups usually found in our children, despite the geographical closeness with Argentina, where the O157 : H7 serotype is prevalent [53]. However, O157 : H7 has been found in Uruguay in a single case of HUS [17], in urinary tract infections of two older patients who did not develop HUS [51] and in multiple food samples [54].

It should be noted that an O96 : H19 EIEC serotype was isolated from two cases without an obvious epidemiological link; this serotype is described as being particularly virulent [55]. Our isolates seemed to be identical, but they require further molecular analysis and comparison with previous regional isolates and with European strains [55–58].

ETEC or EAEC pathotype strains were not found in this group of patients, although they were usually recognized in previous groups of children from Montevideo [13, 14, 16]. In general, ETEC strains are recovered from children who are hospitalized with acute diarrhea and severe dehydration and live in areas with a significant lack of basic services [59]. It does not seem to be the case in our current study. With regard to EAEC, we cannot rule out the participation of atypical strains that do not carry the high molecular weight plasmid (pCVD432). To establish the true role of EAEC strains in diarrheal episodes, we should have performed a screening using the HEp-2 adherence assay or a multiplex PCR targeting plasmid and chromosomal genes. To date, all our EAEC recognized isolates using pCVD432 PCR screening were lysine-decarboxylase positive, which raises doubts about their capacity to cause diarrhea [14, 60].

Antimicrobial treatment is not generally recommended for treatment of diarrheal diseases, with few exceptions. Susceptibility of enteric bacteria should be monitored because resistant genes selected in enteric pathogens or the microbiota can remain undisclosed and be transferred to highly pathogenic microorganisms.

Resistance to the antimicrobial agents was scarce in the DEC isolated in our study, as compared with that observed in previous studies focused on poor children in Montevideo [13]. This fact may result from a general tendency of enteric bacteria in Uruguay towards susceptibility or may simply confirm that the resistance level of bacterial pathogens recovered from towns in the interior of the country is usually lower than that found in the Capital city, where antimicrobial treatment is more widely available and prescribed, contributing to the selection of resistant variants.

Rotavirus infection was observed to be more frequent (14.45%) in the group of children reported here than in another previously studied group (5%) for which vaccination was available [14]. However, groups of children were also different in terms of social parameters and location. Rotavirus vaccine is effective [61] and has been employed in some health services in Uruguay, following WHO recommendation.

The overall proportion of positive etiologic diagnosis was lower (36.14%) in this study than that obtained in a recent similar study (51%) [14], and a limited variety of pathogens was identified. Despite using identical microbiological methods in both studies, delay or difficulties in the sample transport, differences between studied populations, influence of nondeclared previous antibiotic treatment, or other factors may provide additional support to explain a reduced frequency in etiologic diagnosis. However, if appropriate resources and laboratory conditions had been available, investigation of norovirus, usage of CIN for all *Yersinia* cultures, added primers for EAEC PCR, or molecular methods directly applied to feces could have identified a higher proportion and diversity of involved pathogens [11, 62].

## 5. Conclusions

DEC and especially aEPEC are frequently associated with childhood diarrhea in Uruguay.

Atypical EPEC is a presently prevalent pathotype that includes strains closely related to STEC cell lines. Comparative characterization of these bacteria and their molecular relationship or evolution must be performed to provide additional information and data to help support prevention and control.

Animal reservoirs of aEPEC deserve particular attention and further research, considering the close relationship of suburban and rural population with production animals, and taking into account that production and export of food is frequently animal in origin is the main economic activity and income source for Uruguay.

Rotavirus infection is frequent in children throughout the country. Vaccination against this pathogen is an effective health measure that should be extended to all children.

## Figures and Tables

**Table 1:** Primers employed for DEC detection.

Gene	Primer	Sequence 5´-3´	Amplicon Size (bp)	Annealing temperature (°C)	Reference
*eae*	EAE 1	GAGAATGAAATAGAAGTCGT	775	55	[18]
	EAE 2	GCGGTATCTTTCGCGTAATCGCC			

*bfp*	EP1	AATGGTGCTTGCGCTTGCTGC	324	55	[19]
	EP2	GCCGCTTTATCCAACCTGGTA			

*stx*1	VT1-A	GAAGAGTCCGTGGGATTACG	131	55	[20]
	VT1-B	AGCGATGCAGCTATTAATAA			

*stx*2	VT2 a	TTAACCACACCCCACCGGGCAGT	348	55	[20]
	VT2 b	GCTCTGGATGCATCTCTGGT			

*ipa*H	EI1	GTTCCTTGACCGCCTTTCCGATACCGTC	620	55	[21]
	EI2	GCCGGTCAGCCACCCTCTGAGAGTAC			

pCDV 432	EAEC1	CTGGCGAAAGACTGTATCAT	630	60	[16]
	EAEC2	CAATGTATAGAAATCCGCTGTT			

*eltA*	LT-A-1	GGCGACAGATTATACCGTGC	332	55	[16]
	LT-A-2	CCGAATTCTGTTATATATGTC			

*estA*	STA-1	ATTTTTATTTCTGTATTGTCTTT	147	48	[16]
	STA-2	GGATTACAACACAGTTCACAGCAG			

**Table 2:** Clinical findings as related to etiology of diarrhea.

	Children with single identified pathogen^1^ (*n*=27)				Children without identified pathogen^1^ (*n*=53)
	aEPEC (*n*=10)	EIEC (*n*=3)	STEC (*n*=1)	Virus^2^ (*n*=13)	
Children with					
Watery diarrhea	6 (60%)	—	—	10 (76, 9%)	7 (13.2%)
Semiliquid diarrhea	—	—	—	—	28 (52.8%)
Bloody diarrhea	3 (30%)	1 (33.3%)	1 (100%)		—
Mucoid stools	1 (10%)	2 (66.6%)	—	3 (23.1%)	18 (34%)
Abdominal pain	4 (40%)	2 (66.6%)	1 (100%)	6 (46.2%)	6 (11.3%)
Fever	2 (20%)	2 (66.6%)	1 (100%)	5 (35.5%)	5 (9.4%)
Vomiting	1 (10%)	—	—	6 (46.2%)	7 (13.2%)
Fecal leucocytes^3^	1 (10%)	2 (66.6%)	—	—	—

^1^No child was vaccinated against rotavirus at the time of entering to the study; ^2^considering together: rotavirus in 10 children and adenovirus in 3; ^3^significant presence of fecal leucocytes (++ or +++). —, no child showed those conditions.

**Table 3:** Pathotypes and serotypes of recovered DEC isolates in Soriano, Uruguay.

Sample	Serotype	Pathotype	Lactose utilization	Resistant to^*∗*^
V4^**1**^	O166:H21	aEPEC	+	A
V20^**1**^	O137:H6	aEPEC	+	—
V23	O165:H8	aEPEC	+	—
V30	O184:H8	aEPEC	+	A, SAM, CE
V49	O118:H5	aEPEC	+	—
V54	O63:HNT^**2**^	aEPEC	+	CE
V56	O184:H4	aEPEC	+	A, CE, SxT
V61^**1**^	ONT:H−^**3**^	aEPEC	+	A, SxT
V66	O127:H−	aEPEC	+	CE
V74	ONT:H8^**3**^	aEPEC	+	CE
V18^**1**^	O145:H−	STEC	+	A
V48	O96:H19	EIEC	+	—
V73^**1**^	O96:H19	EIEC	+	—

^*∗*^A, ampicillin; CE, cefradine; SAM, sulbactam-ampicillin; SxT, trimethoprim-sulfamethoxazole; —, no resistance traits. ^**1**^Isolates recovered from children with bloody diarrhea. ^**2**^HNT, H-nontypable; ^**3**^ONT, O-nontypable.

## Data Availability

The data used to support the findings of this study are included within the article and are available for further information or requests, on demand.

## References

[1] Fischer Walker C. L., Perin J., Aryee M. J., Boschi-Pinto C., Black R. E. (2012). Diarrhea incidence in low and middle-income countries in 1990 and 2010: a systematic review. *BMC Public Health*.

[2] Liu J., Platts-Mills J. A., Juma J. (2016). Use of quantitative molecular diagnostic methods to identify causes of diarrhoea in children: a reanalysis of the GEMS case-control study. *The Lancet*.

[3] World Health Organization (2013). *Diarrhoeal Disease Fact Sheet 2013*.

[4] Kotloff K. L., Nataro J. P., Blackwelder W. C. (2013). Burden and aetiology of diarrhoeal disease in infants and young children in developing countries (the global enteric multicenter study, GEMS): a prospective, case-control study. *The Lancet*.

[5] Bulled N., Singer M., Dillinghama R. (2014). The syndemics of childhood diarrhoea: a biosocial perspective on efforts to combat global inequities in diarrhoea-related morbidity and mortality. *Global Public Health*.

[6] Ngabo F., Mvundura M., Gazley L. (2016). The economic burden attributable to a child’s inpatient admission for diarrheal disease in Rwanda. *PLoS One*.

[7] Pérez L., Apezteguía L., Piñeyrúa C. (2014). Hemolytic uremic syndrome with mild renal involvement due to Shiga toxin-producing *Escherichia coli* (STEC) O145 strain. *Revista Argentina de Microbiología*.

[8] Pardo L., Mota M. I., Giachetto G., Parada M., Pírez C., Varela G. (2007). Adenitis mesentérica por *Yersinia enterocolitica*. *Revista Médica del Uruguay*.

[9] Humphries R. M., Linscott A. J. (2015). Laboratory diagnosis of bacterial gastroenteritis. *Clinical Microbiology Reviews*.

[10] Cooke M. L. (2010). Causes and management of diarrhoea in children in a clinical setting. *South African Journal of Clinical Nutrition*.

[11] Miliwebsky E., Schelotto F., Varela G., Luz D., Chinen I., Piazza R. M. F., Torres A. G. (2016). Human diarrheal infections: diagnosis of diarrheagenic *Escherichia coli* pathotypes. Chapter 15. *Escherichia coli in the Americas*.

[12] Croxen M. A., Law R. J., Scholz R., Keeney K. M., Wlodarska M., Finlay B. B. (2013). Recent advances in understanding enteric pathogenic *Escherichia coli*. *Clinical Microbiology Reviews*.

[13] Torres M. E., Pírez M. C., Schelotto F. (2001). Etiology of children’s diarrhea in Montevideo, Uruguay: associated pathogens and unusual isolates. *Journal of Clinical Microbiology*.

[14] Varela G., Batthyány L., Bianco M. N. (2015). Enteropathogens associated with community-acquired acute diarrhea in children from households with high socio-economic level. *International Journal of Microbiology*.

[15] Alvarez F., Hormaeche C. E., Demarco R., Alía C., Schelotto F (1974). Consideraciones clínico-bacteriológicas de diarrea aguda de lactantes hospitalizados. (Clinical-Bacteriological considerations about acute diarrhea of hospitalized infants). *Archivos de Pediatría del Uruguay*.

[16] Varela G., Jazisnky C., Gadea P. (2007). Classic Enteropathogenic *Escherichia coli* (EPEC) associated with diarrhea in children users of the Hospital Pereira Rossell. Clinical aspects and characteristics of involved strains. *Revista Medica del Uruguay*.

[17] Gadea M., Varela G., Bernadá M. (2004). Primer aislamiento en Uruguay de *Escherichia coli* productora de toxina Shiga del serotipo O157:H7 en una niña con síndrome urémico hemolítico. (First uruguayan isolate of O157:H7 STEC from a girl undergoing HUS). *Revista Medica del Uruguay*.

[18] Rivas M., Chinen I., Leotta G., Chillemi G. (2011). Manual de Procedimientos. Diagnóstico y caracterización de *Escherichia coli* productor de toxina Shiga O157 y no-O157 a partir de especímenes clínicos. (Diagnostic procedures for investigation of STEC from clinical samples). *OPS-Pan American Health Organization. Servicio Fisiopatogenia, Depto. de Bacteriologia INEI- ANLIS “Carlos G. Malbrán” Argentina*.

[19] Gunzburg S. T., Tornieporth N. G., Riley L. W. (1995). Identification of enteropathogenic *Escherichia coli* by PCR-based detection of the bundle-forming pilus gene. *J Clin Microbiol*.

[20] Pollard D. R., Johnson W. M., Lior H., Tyler S. D., Rozee K. R. (1990). Rapid and specific detection of verotoxin genes in *Escherichia coli* by the polymerase chain reaction. *Journal of Clinical Microbiology*.

[21] Sethabutr O., Venkatesan M., Murphy G. S., Eampokalap B., Hoge C. W., Echeverria P. (1993). Detection of Shigellae and enteroinvasive *Escherichia coli* by amplification of the invasion plasmid antigen H DNA sequence in patients with dysentery. *Journal of Infectious Diseases*.

[22] Blanco M., Blanco J. E., Mora A. (2003). Serotypes, virulence genes, and intimin types of Shiga toxin (verotoxin)-producing *Escherichia coli* isolates from healthy sheep in Spain. *Journal of Clinical Microbiology*.

[23] Clinical and Laboratory Standards Institute (2011). *Performance Standards for Antimicrobial Susceptibility Testing: 21th Informational Supplement M100-S21*.

[24] Trabulsi L. R., Keller R., Tardelli-Gomes T. A. (2002). Typical and atypical enteropathogenic *Escherichia coli*. *Emerging Infectious Diseases*.

[25] Afset J. E., Anderssen E., Bruant G., Harel J., Wieler L., Bergh K. (2008). Phylogenetic backgrounds and virulence profiles of atypical enteropathogenic *Escherichia coli* strains from a case-control study using multilocus sequence typing and DNA microarray analysis. *Journal of Clinical Microbiology*.

[26] Tennant S. M., Tauschek M., Azzopardi K. (2009). Characterization of atypical enteropathogenic *E. coli* strains of clinical origin. *BMC Microbiology*.

[27] Bueris V., Huerta-Cantillo J., Navarro-Garcia F., Ruiz R. M., Cianciarullo A. M., Elias W. P. (2015). Late establishment of the attaching and effacing lesion caused by atypical enteropathogenic *Escherichia coli* depends on protein expression regulated by Per. *Infect Immun*.

[28] Pacheco V. C., Yamamoto D., Abe C. M. (2014). Invasion of differentiated intestinal Caco-2 cells is a sporadic property among atypical enteropathogenic *Escherichia coli* strains carrying common intimin subtypes. *Pathogens and Disease*.

[29] Hu J., Torres A. G. (2015). Enteropathogenic *Escherichia coli:* foe or innocent bystander?. *Clinical Microbiology and Infection*.

[30] Jensen C., Ethelberg S., Olesen B. (2007). Attaching and effacing *Escherichia coli* isolates from Danish children: clinical significance and microbiological characteristics. *Clinical Microbiology and Infection*.

[31] García-Aljaro C., Muniesa M., Blanco J. E. (2005). Characterization of Shiga toxin-producing *Escherichia coli* isolated from aquatic environments. *FEMS Microbiology Letters*.

[32] Bielaszewska M., Kšck R., Friedrich A. W. (2007). Shiga toxin-mediated hemolytic uremic syndrome: time to change the diagnostic paradigm?. *PLoS One*.

[33] Bielaszewska M., Middendorf B., Kšck R. (2008). Shiga toxin-negative attaching and effacing *Escherichia coli*: distinct clinical associations with bacterial phylogeny and virulence traits and inferred in-host pathogen evolution. *Clinical Infectious Diseases*.

[34] Scotland S., Smith H., Cheasty T. (1996). Use of gene probes and adhesion test to caracterize *Escherichia coli* belonging to enteropathogenic serogroups isolated in United Kingdom. *Journal of Medical Microbiology*.

[35] Tobias J., Kassem E., Rubinstein U. (2015). Involvement of main diarrheagenic *Escherichia coli*, with emphasis on enteroaggregative *E. coli*, in severe non-epidemic pediatric diarrhea in a high-income country. *BMC Infectious Diseases*.

[36] Sumbana J., Taviani E., Manjate A., Paglietti B., Santona A., Colombo M. M. (2015). Genetic determinants of pathogenicity of *Escherichia coli* isolated from children with acute diarrhea in Maputo, Mozambique. *Journal of Infection in Developing Countries*.

[37] Nguyen T. V., Le Van P., Huy C., Nguyen Gia K., Weintraub A. (2005). Detection and characterization of diarrheagenic *Escherichia coli* from young children in Hanoi, Vietnam. *Journal of Clinical Microbiology*.

[38] Toledo M. R., Alvariza M. C., Murahovschi J., Ramos S. R., Trabulsi L. R. (1983). Enteropathogenic *Escherichia coli* serotypes and endemic diarrhea in infants. *Infection and Immunity*.

[39] Assis F. E. A., Wolf S., Surek M. (2014). Impact of *Aeromonas* and diarrheagenic *Escherichia coli* screening in patients with diarrhea in Paraná, southern Brazil. *Journal of Infection in Developing Countries*.

[40] Bueris V., Palma Sircili M., Romano Taddei C. (2007). Detection of diarrheagenic *Escherichia coli* from children with and without diarrhea in salvador, Bahia, Brazil. *Memórias do Instituto Oswaldo Cruz*.

[41] Buss S. N., Leber A., Chapin K. (2015). Multicenter evaluation of the BioFire FilmArray gastrointestinal panel for etiologic diagnosis of infectious gastroenteritis. *Journal of Clinical Microbiology*.

[42] Foster M. A., Iqbal J., Zhang C. (2015). Enteropathogenic and enteroaggregative *E. coli* in stools of children with acute gastroenteritis in Davidson County, Tennessee. *Diagnostic Microbiology and Infectious Disease*.

[43] Gómez-Duarte O. G., Arzuza O., Urbina D. (2010). Detection of *Escherichia coli* enteropathogens by multiplex polymerase chain reaction from children’s diarrheal stools in two caribbean–colombian cities. *Foodborne Pathogens and Disease*.

[44] Lozer D. M., Souza T. B., Monfardini M. V. (2013). Genotypic and phenotypic analysis of diarrheagenic *Escherichia coli* strains isolated from Brazilian children living in low socioeconomic level communities. *BMC Infectious Diseases*.

[45] Ochoa T. J., Contreras C. A. (2011). Enteropathogenic *Escherichia coli* infection in children. *Current Opinion in Infectious Diseases*.

[46] Vieira M. A., Gomes T. A. T., Camargo C. H. (2016). Atypical enteropathogenic *Escherichia coli* as etiologic agents of sporadic and outbreak-associated diarrhea in Brazil. *Journal of Medical Microbiology*.

[47] Robins-Browne R. M., Bordun A. M., Tauschek M. (2004). *Escherichia coli* and community-acquired gastroenteritis, Melbourne, Australia. *Emerging Infectious Diseases*.

[48] Martins F. H., Guth B. E. C., Piazza R. M. F. (2016). Lambs are an important source of atypical enteropathogenic *Escherichia coli* in southern Brazil. *Veterinary Microbiology*.

[49] Comery R., Thanabalasuriar A., Garneau P. (2013). Identification of potentially diarrheagenic atypical enteropathogenic *Escherichia coli* strains present in Canadian food animals at slaughter and in retail meats. *Applied and Environmental Microbiology*.

[50] Mota M. I., Gadea M. P., González S. (2010). Bacterial pathogens associated with bloody diarrhea in Uruguayan children. *Revista Argentina de microbiología*.

[51] Gadea M. P., Deza N., Mota M. I. (2012). Two cases of urinary tract infection caused by Shiga toxin-producing *Escherichia coli* O157:H7 strains. *Revista Argentina de microbiología*.

[52] Varela G., Schelotto F. (2015). Síndrome Urémico hemolítico en Uruguay. Aspectos microbiológicos y clínicos, aportes para su conocimiento regional. (Haemolytic-Uremic-Syndrome in Uruguay. Microbiological and clinical aspects; contribution to its regional knowledge). *Revista Facultad de Ciencias de la Salud UDES*.

[53] Pianciola L., D’Astek B. A., Mazzeo M., Chinen I., Masana M., Rivas M. (2016). Genetic features of human and bovine *Escherichia coli* O157:H7 strains isolated in Argentina. *International Journal of Medical Microbiology*.

[54] Varela G., Chinen, Gadea P. (2008). Detection and characterization of Shiga toxin-producing *Escherichia coli* from clinical cases and food in Uruguay. *Revista Argentina de microbiología*.

[55] Escher M., Scavia G., Morabito S. (2014). A severe foodborne outbreak of diarrhoea linked to a canteen in Italy caused by enteroinvasive *Escherichia coli*, an uncommon agent. *Epidemiology and Infection*.

[56] Newitt S., MacGregor V., Robbins V. (2016). Two linked enteroinvasive *Escherichia coli* outbreaks, Nottingham, UK, june 2014. *Emerging Infectious Diseases*.

[57] Toledo M. R., Trabulsi L. R. (1983). Correlation between biochemical and serological characteristics of *Escherichia coli* and results of the Sérény test. *Journal of Clinical Microbiology*.

[58] Chinen I., Rivas M., Caffer M. I., Cinto R. O., Binsztein N. (1993). Diagnosis of Entero-Invasive *Escherichia coli* associated with diarrhea. *Revista Argentina de microbiología*.

[59] Ochoa T. J., Mercado E. H., Durand D. (2011). Frequency and pathotypes of diarrheagenic *Escherichia coli* in Peruvian children with and without diarrhea. *Revista Peruana de Medicina Experimental y Salud Pública*.

[60] Weintraub A. (2007). Enteroaggregative *Escherichia coli*: epidemiology, virulence and detection. *Journal of Medical Microbiology*.

[61] WHO (2013). Rotavirus vaccines. WHO position paper—January (2013). *Weekly Epidemiological Record No. 5*.

[62] Sjölinga Å., Sadeghipoorjahromi L., Novak D., Tobias J. (2015). Detection of major diarrheagenic bacterial pathogens by multiplex PCR panels. *Microbiological Research*.

